# A New Network-Based Strategy for Predicting the Potential miRNA-mRNA Interactions in Tumorigenesis

**DOI:** 10.1155/2017/3538568

**Published:** 2017-08-02

**Authors:** Jiwei Xue, Fanfan Xie, Junmei Xu, Yuan Liu, Yu Liang, Zhining Wen, Menglong Li

**Affiliations:** College of Chemistry, Sichuan University, Chengdu 610064, China

## Abstract

MicroRNA (miRNA) plays an important role in the degradation and inhibition of mRNAs and is a kind of essential drug targets for cancer therapy. To facilitate the clinical cancer research, we proposed a network-based strategy to identify the cancer-related miRNAs and to predict their targeted genes based on the gene expression profiles. The strategy was validated by using the data sets of acute myeloid leukemia (AML), breast invasive carcinoma (BRCA), and kidney renal clear cell carcinoma (KIRC). The results showed that in the top 20 miRNAs ranked by their degrees, 90.0% (18/20), 70.0% (14/20), and 70.0% (14/20) miRNAs were found to be associated with the cancers for AML, BRCA, and KIRC, respectively. The KEGG pathways and GO terms enriched with the genes that were predicted as the targets of the cancer-related miRNAs were significantly associated with the biological processes of cancers. In addition, several genes, which were predicted to be regulated by more than three miRNAs, were identified to be the potential drug targets annotated by using the human protein atlas database. Our results demonstrated that the proposed strategy can be helpful for predicting the miRNA-mRNA interactions in tumorigenesis and identifying the cancer-related miRNAs as the potential drug targets.

## 1. Introduction

MicroRNAs (miRNAs) are a class of endogenous small noncoding RNA molecule with a length of ~22 nucleotides, which regulate gene expression posttranscriptionally [1]. miRNAs can combine with mRNAs to form the RNA-induced silencing complex (RISC) and degrade the mRNAs or inhibit the translation of the target genes [2]. The “seed sequence” with a length of 2 ~ 8 nt at the 5′ end of the miRNA plays an important role in target recognition by binding to the complementary sequences in the untranslated regions (3′-UTRs) of mRNAs [3]. A single miRNA may have the capability to target multiple mRNAs [4, 5] and participates in multiple signaling pathways and biological processes in mammals. It has been reported that miRNAs are involved in numerous cancer-relevant processes such as cell growth, proliferation, apoptosis, migration, and metabolism [6, 7]. The aberrant expression of miRNAs is related to different types of diseases and cancers, such as coronary artery disease [8], gastric cancer [9], lung cancer [10], and breast cancer [11].

Based on the increasing number of studies, miRNAs are being explored as the diagnostic and prognostic biomarkers and as the therapeutic targets for cancer treatment [12]. Previous studies revealed that miRNAs mainly acted as the oncogenic targets or tumor suppressors in the gene regulatory networks [13]. Therefore, two miRNA-based therapeutic strategies were proposed to restore or inhibit miRNA function through miRNA mimics and inhibitors (anti-miRs) [14]. As reported, numerous tumor-suppressive miRNAs and oncogenic miRNAs are promising drug candidates for the treatment of cancers and other diseases [15]. Although most of the miRNA-targeted drugs are still in the preclinical trials, antimiR-122, which is a LNA- (locked nucleic acid-) modified antisense inhibitor, has reached phase II trials for treating hepatitis [16] and the mimics of miR-34, which were encapsulated in lipid nanoparticles, have reached phase I clinical trials for the cancer treatment [17, 18]. Therefore, it is essential to identify the key miRNA candidates for the development of miRNA-based therapeutics of the cancers. In recent years, numerous databases, such as miRBase [19], miRanda [20], DIANA-TarBase [21], and HMDD v2.0 [22], have been developed to investigate the key role of miRNAs in the biological processes and reveal the miRNA-mRNA interaction mechanisms. However, considering the fact that a single miRNA will simultaneously target multiple genes, the miRNA-based therapeutics, which were designed to modulate miRNA expression levels, will affect hundreds of genes. It would be harmful for the patient to randomly regulate the hundreds of transcripts [23]. Thus, it is important to provide an exhaustive analysis of the key miRNAs and the miRNA-mRNA interactions before applying the miRNA-based therapeutics to the clinical trials.

In our study, we proposed a strategy by using the graphical lasso algorithm [24] to discover the key miRNAs and the miRNA-mRNA interaction in tumorigenesis based on the expression levels of miRNAs and mRNAs. A bipartite network with the miRNAs as hubs was constructed to explore the interactions between the miRNAs and mRNAs, and the top 20 miRNAs ranked by their degrees in the network were verified by using three miRNA disease association databases, namely, miRCancer [25], miR2Disease [26], and HMDD v2.0 [22]. Moreover, the gene set enrichment analysis was conducted for the genes that were predicted as the targets in the network by using Database for the Annotation, Visualization, and Integrated Discovery (DAVID) v6.7 [27]. The proposed strategy was validated by using three cancer data sets. Our results showed that for both three data sets, most of the top 20 miRNAs as well as their targeted genes in the network were highly associated with cancers. In addition, the genes, which were predicted to be regulated by more than three cancer-related miRNAs in our study, had been reported as the potential drug targets in previous studies, indicating the satisfactory performance of our proposed strategy on predicting the cancer-related miRNAs and the interactions between miRNAs and their targeted genes.

## 2. Materials and Methods

### 2.1. Datasets

The miRNA expression data, the mRNA expression data, and the clinical data of three types of cancers, namely, acute myeloid leukemia (AML) [28], breast invasive carcinoma (BRCA) [29], and kidney renal clear cell carcinoma (KIRC) [30], were downloaded from the Cancer Genome Atlas (TCGA, https://cancergenome.nih.gov/) data portal. The miRNA-seq data in three data sets were generated by an Illumina Genome Analyzer in the Baylor College Human Genome Sequencing Center (BCGSC). The mRNA-seq data of AML (downloaded on November 7, 2016) were generated by an Illumina Genome Analyzer in the Baylor College Human Genome Sequencing Center (BCGSC). The mRNA-seq data of the BRCA (downloaded on December 15, 2014) and KIRC (downloaded on November 6, 2016) were produced by an Illumina HiSeq 2000 sequencer of the University of North Carolina (UNC). For the three data sets, the read counts for each miRNA and mRNA (data in level 3) were considered the expression level of the miRNA and the mRNA, respectively. In total, we collected 149, 829, and 253 samples for the data sets of AML, BRCA, and KIRC, respectively.

### 2.2. Study Design

**Figure 1:**
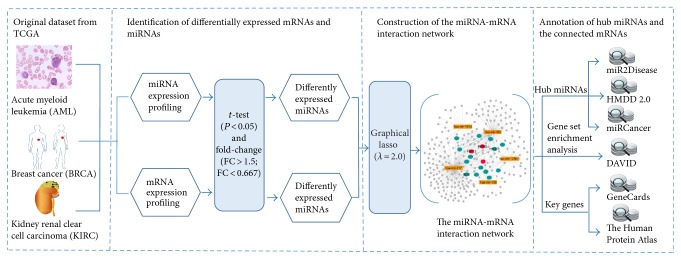
The overview of the study design.

In our study, the graphical lasso algorithm was proposed to construct the miRNA-mRNA interaction network.  Figure 1 showed the overview of our study design. Three cancer data sets, namely, AML, BRCA, and KIRC, were downloaded from the TCGA database, and the differentially expressed miRNA and mRNAs were separately identified for each of the data sets by using the fold change ranking combined with a nonstringent *P* value cutoff. Based on the expression profiles of the differentially expressed miRNAs and mRNAs, the interaction network was constructed by the graphical lasso algorithm, including the connections among the miRNAs and the mRNAs, as well as the connections between miRNAs and mRNAs. The miRNAs and their connected mRNAs in the network were extracted and regrouped into subnetworks, representing the interactions between miRNAs and mRNAs.

To validate whether the cancer-related miRNAs and their key targeted genes can be well characterized by our miRNA-mRNA interaction network or not, we annotated the top 20 miRNAs, which were ranked by their degrees (the number of connections), by using three disease-related miRNA databases, namely, miRCancer [25], miR2Disease [26], and HMDD v2.0 [22], for each of the data sets. Meanwhile, the gene set enrichment analysis was conducted with the targeted genes of the cancer-specific miRNAs by using DAVID v6.7. We checked whether or not the significantly enriched Kyoto Encyclopedia of Genes and Genomes (KEGG) pathways and Gene Ontology (GO) terms were associated with cancers. In addition, we mainly discussed the functions of those genes that were predicted as the targets of more than three miRNAs.

### 2.3. Identification of Differentially Expressed mRNAs and miRNAs

To identify the differentially expressed mRNAs and miRNAs, we firstly divided the samples into two groups for each of the cancer types according to the clinical endpoints. For AML data set, the patients were subdivided into high-risk and low-risk groups according to their survival time. The patients with the survival days longer than one year were assigned to the low-risk group, and the patients with the survival days less than or equal to one year were assigned to the high-risk group. For BRCA data set, the patients were divided into the estrogen receptor- (ER-) positive group and the ER-negative group according to their estrogen receptor status [29]. As to the KIRC data set, the patients in the pathological stages I and II were assigned into the low-risk group and the patients in stages III and IV were assigned into the high-risk group. Then, for all the data sets, Student's *t*-test *P* value was calculated for each of the miRNAs and mRNAs by comparing the expression profiles of the miRNAs and mRNAs between the patient groups. We kept the miRNAs and mRNAs with *P* < 0.05 and calculated the fold changes of them between the compared patient groups, respectively. Finally, the miRNAs and the mRNAs with fold change greater than 1.5 (FC > 1.5) or less than 0.667 (FC < 0.667) were considered the differentially expressed miRNAs and mRNAs, respectively.

### 2.4. Construction of the miRNA-mRNA Interaction Network

As reported, Gaussian graphical models (GGMs) have been widely used to identify the dependent relationship among the variables and to be applied on the biological network inference [31, 32]. In GGMs, the conditional dependence of the two nodes was estimated by an inverse covariance matrix. A nonzero number in the inverse covariance matrix indicates a connection between two nodes [33]. The network inference actually is the estimation of the inverse covariance matrix, and numerous algorithms have been proposed to solve this problem [34]. Notably, based on the GGMs, a more reasonable approach named graphical lasso was proposed to directly estimate a sparse inverse covariance matrix by using the L1 (lasso) penalty [24, 35].

We assume a designed *n* × *m* matrix where *n* indicates the number of samples and *m* is the number of genes or miRNAs. Let ***θ*** = Σ^−1^ and let **S** be the empirical covariance matrix; the problem of estimating ***θ*** is converted to maximize the penalized log-likelihood:

(1) $$\begin{array}{c} \text{log det }\theta -tr\left(S\theta \right)-\rho {\left|\left|\theta \right|\right|}_{1}, \end{array}$$

where tr indicates the trace. ||***θ***||_1_ is the L1 norm of the matrix, which is the maximum value of the sum of the absolute values of the elements in each of the columns in ***θ***, and *ρ* is a nonnegative tuning parameter, which controls the sparseness of the network.

In fact, the graphical lasso gets a ***θ***_*m*×*m*_ matrix to construct the network by using an *n* × *m* matrix as an input. We have two matrices **X**_*n*×*j*_ (*j* miRNA expression profiles of *n* samples) and **Y**_*n*×*k*_ (*k* mRNA expression profiles of *n* samples). Therefore, we integrated these two matrices into the matrix **Z**_*n*×(*j*+*k*)_, which were used to construct an interaction network including the connections among the miRNAs and the mRNAs, as well as the connections between the miRNAs and mRNAs. In our study, only the differentially expressed miRNAs and the mRNAs were used to construct the interaction network and the penalty parameter *ρ* was set to 2.0 for all the data sets. We mainly concentrated on the interactions between the miRNAs and the mRNA in the network.

## 3. Results

### 3.1. Most of the Top 20 miRNAs Were Highly Associated with Cancers

**Figure 2:**
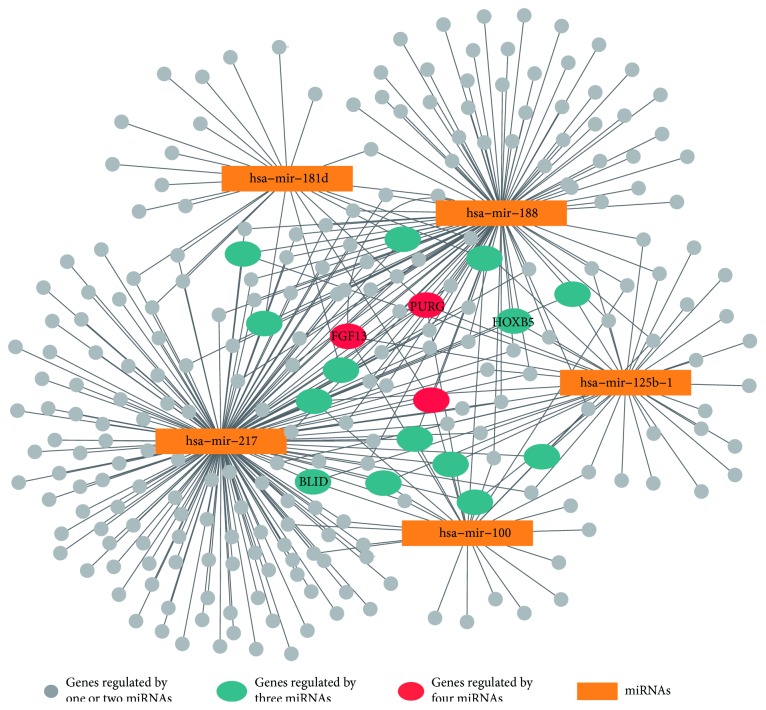
The miRNA-mRNA interaction subnetwork in AML. The five miRNAs in the network were reported to be associated with AML. In the figure, 14 mRNAs (cyan dots) and 3 mRNAs (red dots) were predicted to be connected with three and four miRNAs, respectively. The genes correlated with cancers were marked with their gene symbols.

For AML data set, 34 differentially expressed miRNAs and 798 differentially expressed mRNAs were identified from 706 miRNAs and 20,319 mRNAs, respectively. Considering the miRNAs as the hubs of the miRNA-mRNA interaction network, we selected the top 20 miRNAs ranked by their degrees and listed them in Table 1. It can be seen from the table that 90% (18/20) miRNAs were associated with the cancers after being annotated by the three disease-related miRNA databases. Among the cancer-related miRNAs, five miRNAs, namely, hsa-mir-217, hsa-mir-188, hsa-mir-125b-1, hsa-mir-100, and hsa-mir-181d, were reported to be associated with the acute myeloid leukemia.  Figure 2 showed the subnetworks including these five miRNAs as hubs and their targeted mRNAs.

**Figure 3:**
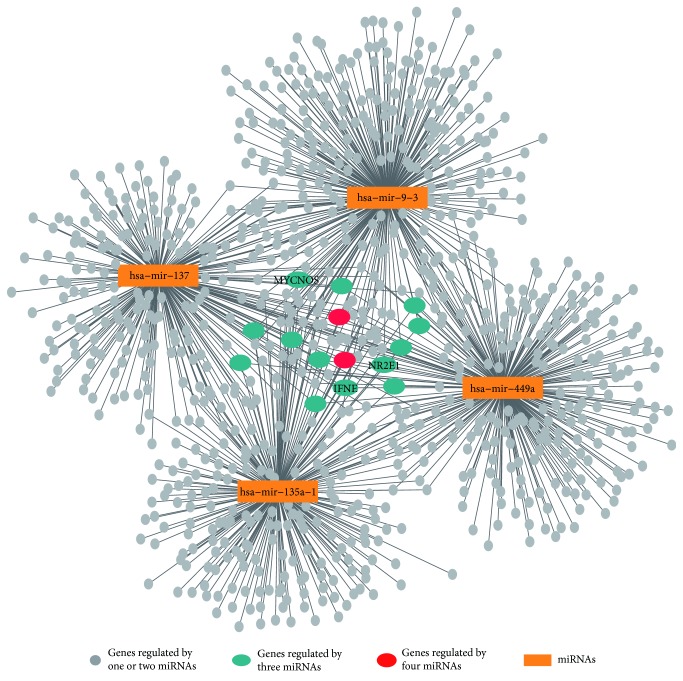
The miRNA-mRNA interaction subnetwork in BRCA. The four miRNAs in the network were reported to be associated with AML. In the figure, 13 mRNAs (cyan dots) and 2 mRNAs (red dots) were predicted to be connected with three and four miRNAs, respectively. The genes correlated with cancers were marked with their gene symbols.

**Figure 4:**
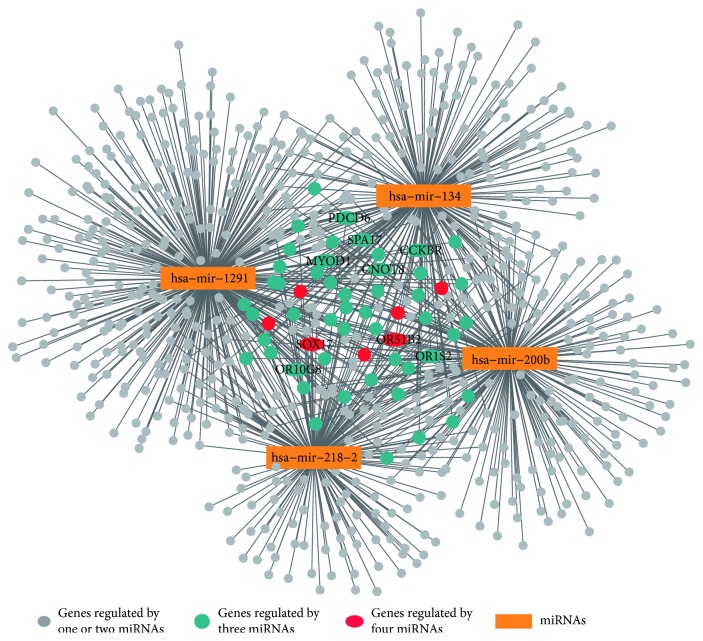
The miRNA-mRNA interaction subnetwork in KIRC. The four miRNAs in the network were reported to be associated with AML. In the figure, 49 mRNAs (cyan dots) and 7 mRNAs (red dots) were predicted to be connected with three and four miRNAs, respectively. The genes correlated with cancers were marked with their gene symbols.

For the data sets of BRCA and KIRC, we identified 266 and 54 differentially expressed miRNAs from 1043 and 1046 miRNAs, respectively, and identified 6021 and 1647 differentially expressed mRNAs from 20,502 and 20,503 mRNAs, respectively. The top 20 miRNAs ranked by their degrees in the miRNA-mRNA interaction network of the BRCA data set were listed in Table 2. It can be seen that 70% (14/20) miRNAs were annotated to be associated with cancers and four out of them, namely, hsa-mir-9-3, hsa-mir-449a, hsa-mir-135a-1, and hsa-mir-137, were breast cancer-specific miRNAs.  Table 3 showed the top 20 miRNAs that were obtained from the interaction network of KIRC. 14 out of 20 (70%) miRNAs were reported to be associated with cancers, and four out of them, namely, hsa-mir-1291, hsa-mir-200b, hsa-mir-134, and hsa-mir-218-2 were directly associated with the renal cell carcinoma. The subnetworks of the specific cancer-related miRNAs and their targeted mRNAs for the data sets of BRCA and KIRC were shown in Figures 3 and 4, respectively.

### 3.2. The mRNAs Targeted by the Cancer-Specific miRNAs Were Significantly Associated with the Biological Process of Cancers

The gene set enrichment analysis was conducted to investigate the gene functions by using the mRNAs, which were predicted as the targets of the cancer-specific miRNAs. For the data sets of AML, BRCA, and KIRC, 255, 853, and 670 targeted mRNAs were used for the gene set enrichment analysis, respectively. The top 5 significantly enriched KEGG pathways were listed in Table 4. For the data sets of AML, BRCA, and KIRC, there were one, five, and two signaling pathways, respectively, which were reported to be associated with cancers. Likewise, the top 5 significantly enriched GO terms related to the biological process and the molecular functions were listed in Table 5. There were four, nine, and eight GO terms for the data sets of AML, BRCA, and KIRC, respectively, which were associated with the tumorigenesis of the cancers.

When focusing on the mRNAs that were predicted to be the targets of multiple miRNAs, we found 14, 13, and 49 mRNAs targeted by three miRNAs in the miRNA-mRNA interaction networks of AML, BRCA, and KIRC, respectively. Moreover, three, two, and seven mRNAs were predicted to be targeted by four miRNAs in the networks of AML, BRCA, and KIRC, respectively. Figures 2, 3, and 4 showed the mRNAs targeted by three miRNAs (cyan dots) and four miRNAs (red dots). We also annotated these genes by using the GeneCards database v4.4.2 (http://www.genecards.org/) and found four, three, and nine genes from the networks of AML, BRCA, and KIRC, respectively, which were reported to be associated with cancers. The HUGO gene symbols of the cancer-related genes were marked in Figures 2, 3, and 4.

## 4. Discussion

In this study, we proposed a new strategy to construct the miRNA-mRNA interaction network based on the expression profiles of miRNAs and mRNAs. The connections between miRNAs and mRNAs were created by the graphical lasso algorithm. We applied the strategy to the three cancer data sets and successfully identified a number of cancer-related miRNAs and their targeted mRNAs.

For the AML data set, 90% miRNAs in the top 20 miRNAs were found to be associated with cancers (Table 1). Among these miRNAs, hsa-mir-100 was considered a potential tumor-related miRNA, which has been reported to regulate cell differentiation by targeting *RBSP3* in acute myeloid leukemia [36]. The pediatric AML patients with the upregulation of miR-100 may have poor relapse-free and overall survival [37]. Moreover, the downregulation of miR-181 family members including miR-181a, miR-181b, miR-181c, and miR-181d was associated with poor prognosis in cytogenetically normal acute myeloid leukemia [38]. For the BRCA data set, 70% miRNAs in Table 2 were associated with cancers and four of them were specifically associated with the breast cancer. has-miR-9 acted as a tumor suppressor, which can inhibit the proliferation of breast cancer cells [39]. miR-137 is a potential tumor suppressor miRNA, which negatively regulates the gene *ERRα* (estrogen-related receptor alpha) by targeting the two functional sites in the 3′-UTR of *ERRα* [40]. As to the KIRC data set, 70% miRNAs in Table 3 were associated with cancers and four of them had been reported to be associated with the development of the renal cell carcinoma. hsa-mir-134 had been reported as a tumor suppressor and can obstruct the tumor growth and metastasis by inhibiting epithelial-mesenchymal transition (EMT) in renal cell carcinoma cells [41]. miR-218 can mediate the focal adhesion pathway and inhibit the cell migration and invasion in renal cell carcinoma [42].

We also inspected the gene functions of the mRNAs, which were predicted to be the targets of the cancer-related miRNAs. The results of gene set enrichment analysis showed that the majority of the KEGG pathways (Table 4) and GO terms (Table 5) were significantly associated with the cancers. In the interaction subnetworks (Figures 2, 3, and 4), several mRNAs targeted by multiple cancer-specific miRNAs were found to have key roles in cancers. For example, the gene *SOX17*, which was predicted to be regulated by four miRNAs in the subnetwork of KIRC, was considered an important tumor suppressor with aberrant methylation for the cancers [43, 44]. In addition, the genes targeted by more than three miRNAs in the subnetworks were mapped to the Human Protein Atlas database v16.1 (http://www.proteinatlas.org), and 8 genes were annotated as the potential drug targets (Table 6).

Note that compared to the conventional drug therapies, the miRNA-targeting drugs have been regarded as a high-value therapy because miRNA may modulate multiple biological processes and pathways. However, there are a lot of challenges for utilizing miRNAs as potential therapeutic targets [45]. The miRNAs regulate tens of thousands of genes which could contribute to both efficacy and unexpected side effects. Therefore, the downstream analysis of genes and pathways regulated by miRNAs should be further elucidated and explored. Due to the complex regulatory mechanisms of the miRNAs, it is still challenging to successfully translate the miRNA-based therapy to the clinics [46]. It is a crucial step in miRNA drug discovery [47] to identify the specific miRNAs as drug targets and clarify the mechanisms of the actions for the key miRNAs. The network-based approach proposed in our study can identify the key miRNAs as well as their targeted mRNAs, which were also significantly associated with the biological process of cancers. It would be helpful for providing the complementary support to the miRNA-targeting drug discovery. In addition, the potential mRNA target could be enriched by integrating the protein structure information and medicinal chemistry. Furthermore, the accumulative information about the side effect and off target relationship from available public resources, such as PharmGKB [48] and the Comparative Toxicogenomics Database (CTD) [49], could be utilized to prioritize the genes regulated by miRNAs for therapeutic target discovery.

## 5. Conclusions

The network-based strategy proposed in our study can efficiently construct the miRNA-mRNA interaction network in tumorigenesis, which included the important cancer-related miRNAs and their targeted genes. The miRNAs and the targeted genes predicted by using the interaction networks may be considered the potential candidates of drug targets in the cancer research.

## Figures and Tables

**Table 1:** The annotation of the top 20 miRNAs in AML.

Cancer type		Number of genes	Disease
AML	hsa-mir-556	170	—
	hsa-mir-217^∗^	163	B-cell chronic lymphocytic leukemia, pancreatic neoplasms, nasopharyngeal carcinoma
	hsa-mir-636	159	Myelodysplastic syndromes, multiple myeloma
	hsa-mir-320c-1	147	Hepatocellular carcinoma, interstitial cystitis
	hsa-mir-639	145	Lung cancer, gastric cancer, breast cancer
	hsa-mir-873	145	Glioblastoma, endometriosis
	hsa-mir-573	138	Pancreatic cancer, esophageal cancer, breast cancer
	hsa-mir-216b	116	Lung neoplasms, nasopharyngeal neoplasms, colorectal neoplasms
	hsa-mir-605	109	Stomach neoplasms, ovarian cancer
	hsa-mir-188^∗^	103	B-cell chronic lymphocytic leukemia, salivary gland neoplasms, rectal neoplasms
	hsa-mir-1468	89	—
	hsa-mir-296	52	Glioma, prostate cancer, urinary bladder neoplasms
	hsa-mir-488	49	Melanoma, ovarian neoplasms, prostatic neoplasms
	hsa-mir-125b-1^∗^	40	Acute myeloid leukemia, breast neoplasms, hepatocellular carcinoma
	hsa-mir-502	36	Colonic neoplasms, ovarian neoplasms, hepatocellular carcinoma
	hsa-mir-551a	32	Stomach neoplasms, ovarian cancer
	hsa-mir-100^∗^	30	Acute myeloid leukemia, precursor cell lymphoblastic leukemia-lymphoma, endometrial neoplasms
	hsa-mir-501	29	Melanoma, atrophic muscular disorders
	hsa-mir-520a	26	Hodgkin's lymphoma, stomach neoplasms, colorectal neoplasms
	hsa-mir-181d^∗^	25	Acute myeloid leukemia, acute promyelocytic leukemia, glioblastoma

^∗^The miRNA was directly associated with AML. ^—^No description of the miRNA was found in the disease-related miRNA database.

**Table 2:** The annotation of the top 20 miRNAs in BRCA.

Cancer type		Number of genes	Disease
BRCA	hsa-mir-1269	381	Lung cancer, colorectal cancer, hepatocellular carcinoma
	hsa-mir-934	368	—
	hsa-mir-2115	325	—
	hsa-mir-618	305	—
	hsa-mir-1251	286	—
	hsa-mir-9-3^∗^	282	Breast neoplasms, stomach neoplasms, glioblastoma
	hsa-mir-105-2	268	Biliary tract neoplasms, hepatocellular carcinoma
	hsa-mir-767	268	Melanoma, rhinitis, allergy, perennial
	hsa-mir-449a^∗^	264	Breast cancer, adenocarcinoma, colonic neoplasms, ovarian neoplasms
	hsa-mir-885	261	Leukemia
	hsa-mir-105-1	253	Biliary tract neoplasms, hepatocellular carcinoma
	hsa-mir-135a-1^∗^	251	Breast neoplasms, colorectal neoplasms, non-small-cell lung carcinoma
	hsa-mir-3662	246	Gastric cancer, head and neck cancer
	hsa-mir-138-1	242	Oral squamous cell carcinoma, renal cell carcinoma, urinary bladder neoplasms
	hsa-mir-376a-2	234	Adrenocortical carcinoma, glioblastoma, lung neoplasms
	hsa-mir-137^∗^	233	Breast neoplasms, malignant melanoma, glioblastoma multiforme
	hsa-mir-3190	232	—
	hsa-mir-138-2	231	Papillary thyroid carcinoma, oral squamous cell carcinoma, pituitary adenoma
	hsa-mir-372	231	Colorectal cancer, acute myeloid leukemia, stomach neoplasms
	hsa-mir-3926-2	231	—

^∗^The miRNA was directly associated with BRCA. ^—^No description of the miRNA was found in the disease-related miRNA database.

**Table 3:** The annotation of the top 20 miRNAs in KIRC.

miRNA	Number of genes	Disease
hsa-mir-1291^∗^	344	Renal cell carcinoma, ovarian cancer, kidney cancer
hsa-mir-558	243	Pancreatic cancer, gastric cancer
hsa-mir-3924	237	—
hsa-mir-376a-1	233	Salivary gland neoplasms, lung neoplasms, adrenocortical carcinoma
hsa-mir-653	229	—
hsa-mir-485	227	Ependymoma, non-small-cell lung carcinoma, leukemia
hsa-mir-200b^∗^	216	Renal cell carcinoma, diabetic nephropathies, pancreatic neoplasms
hsa-mir-134^∗^	215	Renal cell carcinoma, lupus nephritis, glioblastoma
hsa-mir-1246	214	Colorectal neoplasms, esophageal neoplasms
hsa-mir-346	212	Lupus nephritis, hepatocellular carcinoma
hsa-mir-2110	210	Hepatocellular carcinoma, colorectal neoplasms
hsa-mir-365-2	210	—
hsa-mir-153-1	201	Endometrial neoplasms, glioblastoma, rectal neoplasms
‘hsa-mir-374c	191	—
hsa-mir-376b	190	Adrenocortical carcinoma, uterine leiomyoma, epithelial ovarian cancer
hsa-mir-218-2^∗^	184	Renal cell carcinoma, lung cancer, urinary bladder neoplasms
hsa-mir-300	181	Urinary bladder neoplasms, ovarian neoplasms, heart failure
hsa-mir-1303	179	Colorectal neoplasms, hepatocellular carcinoma
hsa-mir-676	174	—
hsa-mir-1237	156	—

^∗^The miRNA was directly associated with KIRC. ^—^No description of the miRNA was found in the disease-related miRNA database.

**Table 4:** The top 5 KEGG pathways enriched with the genes connected with the cancer-specific miRNAs.

Cancer type	KEGG pathways	*P* value
AML	hsa00980: metabolism of xenobiotics by cytochrome	0.0241
	hsa00982: drug metabolism	0.0263
	hsa04740: olfactory transduction^∗∗^	0.0407

BRCA	hsa04080: neuroactive ligand-receptor interaction^∗^	*P* < 0.0001
	hsa00140: steroid hormone biosynthesis ^∗∗^	0.0120
	hsa03320: PPAR signaling pathway^∗∗^	0.0176
	hsa04610: complement and coagulation cascades^∗^	0.0176
	hsa00150: androgen and estrogen metabolism^∗∗^	0.0246

KIRC	hsa05322: systemic lupus erythematosus	0.0003
	hsa04060: cytokine-cytokine receptor interaction^∗∗^	0.0021
	hsa04740: olfactory transduction^∗^	0.0122
	hsa05034: alcoholism	0.0219
	hsa00350: tyrosine metabolism	0.0224

^∗∗^The pathway was directly associated with the corresponding cancer type. ^∗^The pathway was associated with other cancers.

**Table 5:** The top 5 GO terms enriched with the genes connected with the cancer-specific miRNAs.

Cancer type	Category	Term	*P* value
AML	GOTERM_BP_4	GO:0009887 ~ organ morphogenesis	*P* < 0.0001
		GO:0048705 ~ skeletal system morphogenesis	*P* < 0.0001
		GO:0001501 ~ skeletal system development	*P* < 0.0001
		GO:0003002 ~ regionalization^∗∗^	*P* < 0.0001
		GO:0048704 ~ embryonic skeletal system morphogenesis^∗∗^	*P* < 0.0001
	GOTERM_MF_4	GO:0043565 ~ sequence-specific DNA binding^∗∗^	0.0055
		GO:0003700 ~ transcription factor activity	0.0075
		GO:0008236 ~ serine-type peptidase activity	0.0286
		GO:0004888 ~ transmembrane receptor activity^∗^	0.0301

BRCA	GOTERM_BP_4	GO:0019226 ~ transmission of nerve impulse	*P* < 0.0001
		GO:0007268 ~ synaptic transmission^∗∗^	*P* < 0.0001
		GO:0007417 ~ central nervous system development^∗∗^	*P* < 0.0001
		GO:0044057 ~ regulation of system process^∗^	*P* < 0.0001
		GO:0009888 ~ tissue development^∗^	*P* < 0.0001
	GOTERM_MF_4	GO:0030594 ~ neurotransmitter receptor activity^∗∗^	*P* < 0.0001
		GO:0015267 ~ channel activity^∗^	*P* < 0.0001
		GO:0015075 ~ ion transmembrane transporter activity^∗^	*P* < 0.0001
		GO:0008188 ~ neuropeptide receptor activity^∗∗^	*P* < 0.0001
		GO:0005179 ~ hormone activity^∗^	*P* < 0.0001

KIRC	GOTERM_BP_4	GO:0006954 ~ inflammatory response	*P* < 0.0001
		GO:0007186 ~ G protein-coupled receptor signaling pathway^∗∗^	*P* < 0.0001
		GO:0050707 ~ regulation of cytokine secretion^∗^	*P* < 0.0001
		GO:0050663 ~ cytokine secretion^∗^	0.0002
		GO:0050715 ~ positive regulation of cytokine secretion	0.0003
	GOTERM_MF_4	GO:0005125 ~ cytokine activity^∗^	0.0002
		GO:0004930 ~ G protein-coupled receptor activity^∗∗^	0.0002
		GO:0001664 ~ G protein-coupled receptor binding^∗∗^	0.0004
		GO:0005126 ~ cytokine receptor binding^∗^	0.0007
		GO:0004984 ~ olfactory receptor activity^∗^	0.0013

^∗∗^The Go term was directly associated with the corresponding cancer type. ^∗^The Go term was associated with other cancers.

**Table 6:** The annotation of the key genes connected with more than three cancer-specific miRNAs in the miRNA-mRNA interaction networks.

Cancer type	Gene	Gene description	Protein class
AML	ASPG	Asparaginase	Enzymes, predicted intracellular proteins

BRCA	AQP2	Aquaporin 2 (collecting duct)	Disease-related genes, potential drug targets, predicted membrane proteins, transporters

KIRC	CNOT8	CCR4-NOT transcription complex subunit 8	Enzymes, plasma proteins, predicted intracellular proteins
	CTPS1	CTP synthase 1	Disease-related genes, enzymes, potential drug targets, predicted intracellular proteins
	IFNAR2	Interferon (alpha, beta, and omega) receptor 2	Cancer-related genes, FDA-approved drug targets, predicted intracellular proteins, predicted membrane proteins
	MOCS2	Molybdenum cofactor synthesis 2	Disease-related genes, enzymes, potential drug targets, predicted intracellular proteins
	PRSS37	Protease, serine 37	Enzymes, predicted secreted proteins
	VCP	Valosin-containing protein	Disease-related genes, enzymes, plasma proteins, potential drug targets, predicted intracellular proteins, transporters
